# The course of gait speed during a 400m walk test of mobility limitations in community-dwelling older adults

**DOI:** 10.1007/s00391-021-01908-5

**Published:** 2021-06-11

**Authors:** Ulrich Lindemann, Sebastian Krumpoch, Clemens Becker, Cornel C. Sieber, Ellen Freiberger

**Affiliations:** 1 Department of Clinical Gerontology and Rehabilitation, Robert-Bosch-Hospital, Stuttgart, Germany; 2 Institute for Biomedicine of Aging (IBA), Friedrich-Alexander-University of Erlangen-Nürnberg (FAU), Nuremberg, Germany; 3 Department of Internal Medicine, Kantonsspital Winterthur, Winterthur, Switzerland

**Keywords:** Cross-sectional studies, Geriatric assessment, Humans, Walking, Aging, Querschnittsstudien, Geriatrisches Assessment, Menschen, Gehen, Altern

## Abstract

**Background:**

The 400‑m walk test (400MWT) of usual gait speed is an assessment of mobility limitations in geriatric medicine and sarcopenic research.

**Objective:**

The aim of this study was to describe the course of gait speed during a 400MWT in community-dwelling older adults in terms of physical, psychological and general health-related outcomes. Possible plateau phases during the 400MWT could enable integrated measurements of short distance walk tests.

**Methods:**

In this study 148 community-dwelling older adults (mean age 80.4 ± 4.4 years, 61% women) performed a 400MWT at comfortable gait speed. Additionally, an 8m walk test was carried out and history of falling, sex, comorbidities, fear of falling, executive function and gait variability were determined as covariates.

**Results:**

Gait speed was higher in the beginning and the end of the 400MWT compared to the middle part with respect to all analyzed covariates. Mean gait speed of the 8 m walk test was significantly faster than mean gait speed of the 400MWT (*t *(df = 147) = 0.07,* p* = 0.001).

**Conclusion:**

The course of gait speed during a 400MWT performed by community-dwelling older adults was not affected by sex, gait variability, comorbidity, history of falling, fear of falling or executive function. Gait speed measurements of the 400MWT do not fully represent assessment of supervised short distance gait speed in community-dwelling adults.

## Abstract (de)

**Hintergrund:**

Der 400 m-Gehtest (400MWT) in habitueller Ganggeschwindigkeit erfasst Mobilitätseinschränkungen in der Geriatrie und Sarkopenieforschung.

**Ziel:**

Ziel der Studie war es, den Verlauf der Ganggeschwindigkeit während eines 400MWT bei selbstständig lebenden älteren Erwachsenen in Abhängigkeit von physischen, psychologischen und allgemein gesundheitsbezogenen Faktoren zu beschreiben. Mögliche Plateauphasen während des 400MWT könnten integrierte Messungen kurzer Gehtests ermöglichen.

**Methode:**

Insgesamt 148 selbstständig lebende ältere Erwachsene (Durchschnittsalter 80,4 ± 4,4 Jahre, 61 % Frauen) absolvierten einen 400MWT in habitueller Ganggeschwindigkeit. Zusätzlich wurde ein 8 m-Gehtest durchgeführt, daneben wurden die Parameter Sturzereignisse, Geschlecht Komorbidität, Angst vor Stürzen, exekutive Funktionen und Gangvariabilität als Kovariaten bestimmt.

**Ergebnisse:**

Die Ganggeschwindigkeit im 400MWT war hinsichtlich aller analysierten Kovariaten anfangs und am Ende höher als im mittleren Teil. Die durchschnittliche Ganggeschwindigkeit im 8 m-Gehtest war signifikant höher als im 400MWT (*t *(df = 147) = 0,07, *p* = 0,001).

**Schlussfolgerung:**

Der Verlauf der Ganggeschwindigkeit während eines 400MWT bei selbstständig lebenden älteren Erwachsenen wurde nicht von den Kovariaten Geschlecht, Gangvariabilität, Komorbidität, Sturzereignisse, Sturzangst oder exekutive Funktionen beeinflusst. Ganggeschwindigkeitsmessungen des 400MWT sind nur bedingt auf supervidierte, kürzere Assessments der Ganggeschwindigkeit bei selbstständig lebenden Erwachsenen generalisierbar.

## Introduction

Long-distance walk tests, when performed as fast as possible, are widely used to assess cardiorespiratory fitness. In contrast, the 400‑m walk test (400MWT) with the instruction to walk with usual pace is often incorporated into epidemiological studies to measure mobility limitations [1–3]. Compared to other functional outcome measures such as the short physical performance battery (SPPB), it is less prone to ceiling effects in high-functioning older adults [4] and mean usual gait speed can be calculated from completion time.

Habitual/usual gait speed is increasingly recognized as a vital sign [5]. Gait speed slower than 1.0 m/s has been predictive of negative outcomes, like frailty, mortality, mobility limitations, falls and decreased quality of life [6–8]. Therefore, shortdistance walk tests over 4m, 8m or 10m, when performed at usual pace, are commonly used to describe a person’s general physical ability [9, 10]. Assessment protocols covering several research questions can be overdemanding and should be merged whenever possible. Intuitively, the assessment of a shortdistance walk to assess gait speed and a long-distance walk could be combined if both tests are performed with the same pace instructions. Measurements of usual walking have been shown to be affected by various factors (e.g. distance, acceleration/deceleration phase, instructions, measurement instruments), which should therefore be standardized [5]. For instance, Najafi et al. (2009) found a significant increase in stride time velocity during long walking distances (e.g. > 20m) compared to short walking distances (e.g. < 10 m) in older persons [11]. The course of gait speed during even longer distances has not been investigated so far. It is therefore questionable whether 400MWT generally show plateau phases (e.g. start, middle, end) that would allow integration of a shortdistance walk test.

In addition, a person’s walking performance is modified by individual characteristics [12], such as physical, psychological and/or general health aspects. Accordingly, the performance of functionally limited persons during a 400MWT should be more affected by factors like fatigue, stability/regularity of walking [12, 13], executive function, fear of falling [14, 15], comorbidity and falls history. In contrast, healthy older adults should be more influenced by motivational factors resulting in overperforming or initial/final sprint.

The aim of this study was to describe the course of gait speed during a 400MWT of usual walking pace in community-dwelling older adults and to determine whether a possible plateau phase of gait speed would allow integration of a shortdistance walk test. In addition, we investigated the impact of physical, psychological, and general health-related covariates on the course of gait speed during a 400MWT.

## Material and methods

### Subjects and design

In total 148 community-dwelling older adults from the greater area of a south-east German city were assessed between May and December 2019. The participants were recruited via an existing data pool and distribution of flyers. Inclusion criteria were an age of at least 70 years, living independently, the ability to walk 400m and the ability to follow instructions. Participants were excluded if they were dependent on the use of wheeled walkers and/or reported serious orthopedic and/or neurologic diseases causing gait impairments. Assessments lasted approximately 120–240 min and consisted of a structured personal interview and a gait analysis, including a 400MWT, in a well-lit hallway. The study protocol was approved by the ethics committee of the Friedrich Alexander University (43_19B) and all participants gave written informed consent.

### Primary outcome

**Fig. 1:**
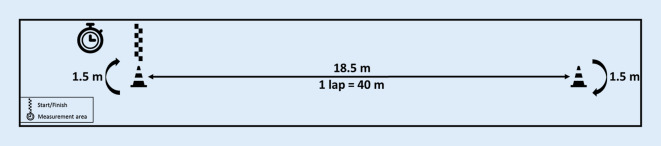
Start/finish at the left cone

The 400MWT was performed according to an evaluated protocol to assess mobility limitations [2]. This test consisted of 10 laps over a 20 m course marked by two traffic cones, placed 18.5 m apart, as an average turning distance of 1.5m was assumed (shown in Fig. 1). Participants started from a standing position at the left cone and walked up and down the corridor in a continuous loop, passing the course twice in each lap. The objective was to complete the 400m at a usual, preferably steady, pace but without overexertion. The instruction was as follows: “This is not a fitness test. Please walk at a speed as if you were taking a stroll in the park, knowing that you have a longer distance to cover.” To increase ecological validity, participants were asked to wear their own habitual outdoor shoes and use their assistive devices. Only wheeled walkers, which probably would have caused problems during the turning phases of the 400m walk test, were not permitted. Participants paused to rest whenever necessary and the number of rests, taken while standing without touching the walls, was recorded. The test was aborted at any time if requested or at signs of overexertion. Total time of the 400MWT and each individual lap were assessed by stopwatch and mean gait speed was calculated. In the case of noncompletion, gait speed was obtained from the distance and time walked until drop-out.

### Descriptive parameters/covariates

Demographic and anthropometric data, such as age, sex, body weight and height were recorded. With respect to psychological parameters, depressive symptoms were assessed using the 15-item geriatric depression scale (GDS) [16]. The scores ranged from 0 to 15, with greater values indicating greater depressive symptoms. The Montreal cognitive assessment (MoCA) measured global cognitive function with a maximum/best score of 30 [17]. Previous studies have demonstrated a relationship between psychological outcomes, such as executive function, and gait abilities of older adults [15]. Therefore, executive function was measured by the trail making test (TMT) [18] consisting of a part A (visuomotor tracking) and a part B (visuoconceptual function). The difference between the two completion times (TMT delta) was taken as outcome. The falls efficacy scale-international (FES‑I), a 16-item questionnaire with a maximum/worst score of 64 [19] was performed. Fear of falling is prevalent among older adults, affects gait performance and is a proven predictor of fall risk [14].

Regarding general health, comorbidity was evaluated using the functional comorbidity index (FCI), a list of 18 comorbid diseases that are associated with physical function [20]. Additionally, history of falling was obtained, with “fallers” defined as individuals who reported at least one fall during the last year.

In terms of physical parameters, global physical function of the participants was described using the SPPB, which included a repeated sit-to-stand transfer, a static balance test and a 4m usual paced walk test [21]. Each test component of the SPBB had a possible score of 0–4. Total scores ranged from 0 to 12, with higher values indicating better physical function. Furthermore, gait speed, stride length variability and step width variability, indicating capacity and quality/regularity of walking [12], of all participants were assessed on an 8m long instrumented walkway with embedded pressure sensors (GAITRite, Franklin, NJ, USA) with additional 2.5m for acceleration and deceleration before and behind the mat [22, 23]. Gait variability quantifies fluctuations in temporal and spatial walking patterns and is an indirect measure of gait stability and regularity [24, 13, 12]. Following a standardized protocol, participants were instructed to walk with their usual pace and their own outdoor shoes.

Assessment instruments showing floor or ceiling effects were not considered as covariates for subgroup analyses (shown in Tables 1 and 2). Furthermore, age was also excluded due to its narrow range and high association with the other covariates.

**Table 1:** Descriptive parameters, which partly served as covariates (^a^), of all (*n* = 148) participating older adults

Descriptive parameters/covariates^a^	Mean ± SD (range) or *n* (%)
Sex, female/male^a^	90 (60.8)/58 (39.2)
Age (years)	80.4 ± 4.4 (71–93)
Body height (cm)	164.0 ± 10.4 (142–198)
Body weight (kg)	75.0 ± 16.3 (43.9–121.9)
Body mass index (kg/m^2^)	27.8 ± 4.9 (17.6–43.3)
Short physical performance battery (0–*12*)	11.0 ± 1.6 (4–12)
Functional comorbidity index (*0*–18)^a^	3.6 ± 2.2 (0–9)
Fallers, yes/no^a^	55 (37.2)/93 (62.8)
Falls efficacy scale – international (*16*–64)^a^	21.1 ± 5.7 (16–44)
Geriatric depression scale (*0*–15)	1.69 ± 2.0 (0–10)
Montreal cognitive assessment (0–*30*)	25.4 ± 2.9 (13–30)
Trail making test delta (s)^a^	86.2 ± 51.7 (12–266)
400 m walking aid, yes/no	19 (12.8)/129 (87.2)
400‑m completion, yes/no	144 (97.3)/4 (2.7)
400‑m rest, yes/no	9 (6.1)/139 (93.9)
8‑m gait speed (m/s)	1.21 ± 0.3 (0.33–1.80)
400‑m gait speed (m/s)	1.14 ± 0.3 (0.26–1.74)
8‑m stride length variability (%)^a^	2.97 ± 1.5 (0.8–8.6)
8‑m step width variability (%)^a^	27.05 ± 14.2 (7.1–78.2)

Better score values are italicized, *SD* standard deviation, *n* number of cases

**Table 2:** Mean gait speed during the 400m walk test for all subgroups

Covariates *(Dichotomized variables)*	*N*	Gait speed [m/s], Mean (95% confidence interval)
Gender, female/male	90/58	1.11 (1.11–1.12)/1.19 (1.18–1.20)
Step width variability, ≤ 27%/> 27%^*a*^	96/52	1.12 (1.11–1.13)/1.18 (1.18–1.19)
Stride length variability, ≤ 2.86%/> 2.86%^*b*^	83/65	1.22 (1.21–1.22)/1.05 (1.04–1.06)
Falls, yes/no	55/93	1.10 (1.09–1.11)/1.17 (1.16–1.18)
Functional comorbidity index, ≤ 3/> 3	75/73	1.24 (1.23–1.24)/1.04 (1.03–1.06)
Falls efficacy scale – international, ≤ 22/> 22^*c*^	109/39	1.21 (1.20–1.22)/0.94 (0.93–0.95)
Trail making test delta, < 72 s/≥ 72 s^*d*^	73/74	1.18 (1.17–1.18)/1.11 (1.10–1.12)

^a^Ciprandi et al. (2017) [24]

^b^Doi et al. (2020) [26]

^c^Delbaere et al. (2010) [25]

^d^Hobert et al. (2011) [27]

### Statistics

Variables were described by mean, standard deviation and 95% confidence interval, minimum and maximum. For subgroup analysis the results of covariates were dichotomized to high versus low performers regarding reference values [24–27], median split (comorbidity) or obvious categories (sex, falls). A paired t‑test was performed to analyze the difference between the gait speed of the 400MWT and gait speed of the 8m walk test. All analyses were performed using SPSS® version 24.0 (SPSS, Inc., Chicago, IL, USA).

## Results

**Fig. 2:**
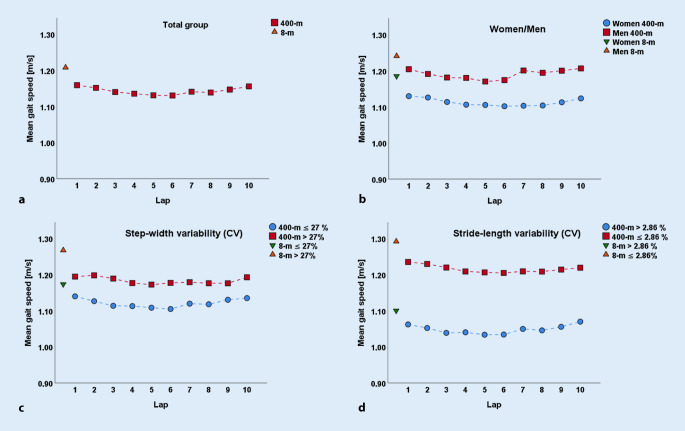
Gait speed during the 400m walk test and 8m walk test for (**a**) the total group, and the covariates (**b**) women and men, **c** step width variability^a^ ≤ 27% and > 27% and **d** stride length variability^b^ ≤ 2.86% and > 2.86%. ^*a,b*^*Assessed with GAITRite system*

**Fig. 3:**
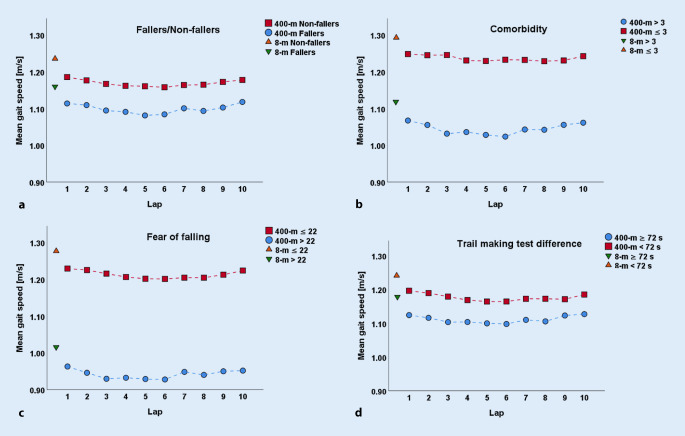
Gait speed during the 400m walk test and 8m walk test for the covariates **a** fallers and non-fallers, **b** comorbidity ≤ 3 and > 3, **c** fear of falling ≤ 22 and > 22 and **d** trail making test difference < 72 s and ≥ 72 s

In this study 148 participants with a mean age of 80 years (61% women) were recruited of whom 144 (97%) completed the 400MWT and 19 (13%) walked with a walking aid. The SPPB (81 participants with maximum score), the GDS (90 participants with minimum score) and the MoCA (79 participants with a score of > 25) showed ceiling effects and were therefore not considered for subgroup analysis (meaning dichotomization as high and low performers). Descriptive parameters of all participants are presented in detail in Table 1. The mean gait speed of all dichotomized variables over all 10 laps of the 400MWT is described in Table 2. Differences in gait speed between low and high performers were shown, as confidence intervals did not overlap for any covariate analyzed. A general pattern of gait speed during the 400MWT was observed that was similar between men and women and for all other subgroups (shown in Fig. 2 and 3). Gait speed peaked in the first lap, then dropped to a relatively stable plateau phase in the middle part of the 400m and increased again through the end of the test. Furthermore, mean gait speed of the 8m walk test was significantly faster than mean gait speed of the 400MWT (*t *(df = 147) = 0.07,* p* = 0.001).

## Discussion

Our results present a general pattern of gait speed during a 400MWT in a relatively fit population of community-dwelling older adults who are unaffected by physical, psychological, and general health-related outcomes. In detail, this course of gait speed was shown to be independent of the influence of the covariates sex, gait variability, fear of falling, executive function, comorbidity or falls history. A relatively stable plateau phase of gait speed was observed in the midsection of the 400MWT. With respect to steady state gait speed measurements, our results imply that a shortdistance walk test of usual gait speed could be included into this middle part of the 400m; however, since a paired t‑test showed a significant difference between the walking speeds of the 400MWT and an 8m walk test, this recommendation can only be issued with reservations.

It is noteworthy that the gait speed in each lap of the 400MWT was slower than in the shortdistance walk test. This could be explained by fatigue and/or several decelerations during the turning phases around the cones. Furthermore, walking over a longer distance may reduce the awareness of being observed. It has been shown that supervised gait speed measurements in a laboratory were faster than unsupervised gait speed assessed during daily life in community-dwelling adults [28]. This suggestion is corroborated by the fact that gait speed decreased after the start over 2–3 laps in our study. Future studies should therefore compare gait speed of the middle part of a 400MWT with daily life unsupervised gait speed.

Our results contrast with the abovementioned publication of Najafi et al. [11], which examined only a small sample (*n* = 24) of older adults. It could be argued that their long walks (20 m) were much shorter than the 400MWT and thus were not longdistance walks. Furthermore, our results are corroborated by Pasma et al. who found that gait speed of a 10m walk was faster than velocities measured during a 6-min walk, although the persons were instructed to walk as fast as possible during this longdistance walk [29]. Nevertheless, our results cannot be applied to longdistance walk tests, such as the 2‑min, 6‑min [30], 12-min [31] or the 400MWT [32], if instructed to walk as far or as fast as possible over the given time or distance in order to assess cardiorespiratory fitness. Here, a different course of gait speed could be possible. As a limitation of our study a participation of mainly physical healthy older adults has occurred due to the broad inclusion criteria affecting the generalizability of the study.

## Conclusion

In conclusion, the general pattern of decrease, plateau and increase of gait speed during a 400MWT is similar for high and low performers. Furthermore, the course of gait speed during a long-distance walk tests performed by community-dwelling older adults in usual pace is not modified by sex, gait variability, comorbidity, history of falling, fear of falling or executive function. Since the awareness of being observed is probably higher at the beginning and the end of long-distance measurements, the middle part of the 400MWT might correspond to unsupervised gait speed during daily life in community-dwelling older adults. Future studies should investigate this.

## Relevance to clinical practice

- The course of gait speed during a 400MWT performed by community-dwelling older adults is not modified by sex, regularity of walking, comorbidity, history of falling, fear of falling or executive function.
- Mean gait speed of an 8m short-distance walk test was significantly faster than mean gait speed of the 400MWT.

## Declarations

The study was performed in accordance with the ethical standards of the ethics committee of the local university and with the 1964 Helsinki declaration and its later amendments. This article does not contain any studies with animals performed by any of the authors. This study was approved by the Friedrich-Alexander-University ethics committee (43_19B). All participants of this project gave written informed consent for data analysis and publication.
